# ImmuNet: a segmentation-free machine learning pipeline for immune landscape phenotyping in tumors by multiplex imaging

**DOI:** 10.1093/biomethods/bpae094

**Published:** 2024-12-20

**Authors:** Shabaz Sultan, Mark A J Gorris, Evgenia Martynova, Lieke L van der Woude, Franka Buytenhuijs, Sandra van Wilpe, Kiek Verrijp, Carl G Figdor, I Jolanda M de Vries, Johannes Textor

**Affiliations:** Medical BioSciences, Radboudumc, Nijmegen 6562 GA, The Netherlands; Data Science Group, Institute for Computing and Information Sciences, Radboud University, Nijmegen 6525 EC, The Netherlands; Medical BioSciences, Radboudumc, Nijmegen 6562 GA, The Netherlands; Oncode Institute, Radboudumc, Nijmegen 6525 GA, The Netherlands; Medical BioSciences, Radboudumc, Nijmegen 6562 GA, The Netherlands; Data Science Group, Institute for Computing and Information Sciences, Radboud University, Nijmegen 6525 EC, The Netherlands; Medical BioSciences, Radboudumc, Nijmegen 6562 GA, The Netherlands; Oncode Institute, Radboudumc, Nijmegen 6525 GA, The Netherlands; Department of Pathology, Radboudumc, Nijmegen 6525 GA, The Netherlands; Data Science Group, Institute for Computing and Information Sciences, Radboud University, Nijmegen 6525 EC, The Netherlands; Medical BioSciences, Radboudumc, Nijmegen 6562 GA, The Netherlands; Department of Medical Oncology, Radboudumc, Nijmegen 6525 GA, The Netherlands; Oncode Institute, Radboudumc, Nijmegen 6525 GA, The Netherlands; Department of Pathology, Radboudumc, Nijmegen 6525 GA, The Netherlands; Medical BioSciences, Radboudumc, Nijmegen 6562 GA, The Netherlands; Oncode Institute, Radboudumc, Nijmegen 6525 GA, The Netherlands; Medical BioSciences, Radboudumc, Nijmegen 6562 GA, The Netherlands; Medical BioSciences, Radboudumc, Nijmegen 6562 GA, The Netherlands; Data Science Group, Institute for Computing and Information Sciences, Radboud University, Nijmegen 6525 EC, The Netherlands

**Keywords:** cell detection, click annotations, deep learning, multiplex immunohistochemistry

## Abstract

Tissue specimens taken from primary tumors or metastases contain important information for diagnosis and treatment of cancer patients. Multiplex imaging allows *in situ* visualization of heterogeneous cell populations, such as immune cells, in tissue samples. Most image processing pipelines first segment cell boundaries and then measure marker expression to assign cell phenotypes. In dense tissue environments, this segmentation-first approach can be inaccurate due to segmentation errors or overlapping cells. Here, we introduce the machine-learning pipeline “ImmuNet”, which identifies positions and phenotypes of cells without segmenting them. ImmuNet is easy to train: human annotators only need to click on an immune cell and score its expression of each marker—drawing a full cell outline is not required. We trained and evaluated ImmuNet on multiplex images from human tonsil, lung cancer, prostate cancer, melanoma, and bladder cancer tissue samples and found it to consistently achieve error rates below 5%–10% across tissue types, cell types, and tissue densities, outperforming a segmentation-based baseline method. Furthermore, we externally validate ImmuNet results by comparing them to flow cytometric cell count measurements from the same tissue. In summary, ImmuNet is an effective, simpler alternative to segmentation-based approaches when only cell positions and phenotypes, but not their shapes, are required for downstream analyses. Thus, ImmuNet helps researchers to analyze cell positions in multiplex tissue images more easily and accurately.

## Introduction

Tissue samples provide key information about the manifestation and progression of many diseases. In clinical oncology, histopathological examinations serve as an important basis for cancer diagnosis, treatment response monitoring, and relapse detection. There are also intensive ongoing efforts to develop histological biomarkers for selecting the appropriate treatment for cancer patients. Traditionally, tissue specimens are evaluated manually by trained pathologists, but machine learning (ML) systems are being developed to automate some aspects of tissue evaluation and improve the objectivity, reproducibility, and scalability of these aspects of histopathology [1].

A core task of histopathological analysis is the localization of different types of cells. Many types of cells, such as epithelial cells and cancer cells, can be accurately identified based on morphological aspects like size, shape, or nuclear atypia. For applications in cancer immunotherapy, accurate identification of immune cell subsets is especially important, as different immune cell subtypes perform very different functions within the tumor microenvironment. Immune cell subsets often differ very little in morphology and need to be identified based on the expression of marker proteins. Such cells come in many flavors that require combinations of multiple markers for proper identification; for instance, T cells alone can be grouped in up to 10 major subsets, several of which can be subdivided further [2]. To allow *in situ* mapping of immune cell subsets, several multiplex imaging techniques have been developed, such as multiplex immunohistochemistry (mIHC) [3], co-detection by indexing (CODEX) [4], cytometry by time of flight (CyTOF) [5], or NanoString’s digital spatial profiling [6]. All these techniques deliver multi-channel images that typically consist of a nuclear stain (such as DAPI) together with nuclear, cytoplasm, or membrane markers to identify cell locations and phenotypes.

Conventionally, cell phenotyping in multiplex image analysis is seen as a downstream task of cell segmentation [7]. A simple approach is to use cell segmentation masks to measure the brightness in each channel. This results in short, interpretable vectors of expression intensities for each cell, which can be further processed by clustering or manual thresholding. Cell segmentation is a challenging problem with a long history and is well-studied in biomedical imaging [8–10]. Nowadays, traditional approaches to cell segmentation, such as the Watershed algorithm [11], have been largely replaced by end-to-end ML [12–14]. Human-level performance in segmentation has been unlocked through the construction of TissueNet, a dataset of over one million labeled cells with both nuclei and whole-cell segmentation across diverse imaging platforms and tissues [15]. When trained on TissueNet, the state-of-the-art cell segmentation algorithms Cellpose [16], StarDist [17], and Mesmer [15] achieved equivalent performance that is on par with that of expert human annotators. However, the existence of truly accurate instance segmentation remains questionable due to an overlap between adjacent cells, which is common at high tissue densities even in thinly cut slices [18]. Unless a segmentation algorithm outputs cell masks that can overlap around the cell boundaries, even high-quality segmentation remains inherently inaccurate. Further issues that complicate the analysis of segmented mIHC data include channel crosstalk, where a signal from one channel is partially present in another channel; steric hindrance, where cells that are already stained with one antibody may become less efficient targets for subsequent antibodies [3]; and signal spillover, when adjacent cells overlap [19].

Due to such complexities, simple clustering or thresholding approaches for segmentation-based phenotyping of mIHC data require several labor-intensive rounds of manual adjustment to achieve satisfactory results [20–24], if these are even achievable. This can be mitigated by using methods that correct for signal spillover [4, 25] and channel crosstalk [26], extracting marker expression profiles using clusters of pixel-level features instead of raw image channels [18], or leveraging information about phenotypes in the cell neighborhood [27]. Alternatively, the phenotyping problem can be treated as another subsequent ML step, such as the recently proposed CellSighter [19]. This plethora of approaches can result in complex multi-stage pipelines that are difficult to implement, optimize, and maintain, and carry the risk of error propagation.

In this article, we aim to develop an ML approach for cell localization and phenotyping that is as simple to train and run as possible, while still delivering decent accuracy. Two core ideas underpin our approach. First, we dispense with the cell segmentation step and limit ourselves to detecting the positions of cells. This is based on our observation that many studies do not actually need the cell shape information to answer their research questions; for example, tumor microenvironment analyses often focus on cell densities in different spatial compartments such as the tumor or the invasive margin [28–33]. By ignoring the shapes of cells, we not only simplify the problem itself but also reduce the amount of work needed to collect annotations, which can now simply be clicks on the centers of cells. Second, we combine cell detection and phenotyping into a single, multi-task architecture. The multi-task learning approach to object detection and classification has proven to be faster, more accurate, and more generalizable than its multi-stage counterparts [34, 35]. Multi-task learning has also already been proven effective for both histopathology images [36, 37] and single-stain immunohistochemistry [38, 39].

Altogether, we propose a simple and fast *segmentation-free* pipeline that treats cell localization and phenotyping as an integrated problem, requires minimal post-processing, and can be trained on sparse point annotations, which are considerably less time-consuming to collect than fully annotated regions. Our approach is much simpler to implement and optimize than traditional segmentation-based pipelines, yet it achieves accurate results. To allow for some flexibility in downstream analysis and definition of phenotypes, we predict a probability of expression for each cellular marker instead of directly predicting the phenotype. We opt for a fully convolutional architecture that allows fast inferencing on full multiplex images of varying sizes, but we note that the overall setup can be combined with other backbone architectures.

## Materials and methods

### Ethics approval and consent to participate

The study of the melanoma material collected at the Radboudumc was officially deemed exempt from medical ethical approval by the local Radboudumc Medical Ethical Committee concurrent with Dutch legislation, as we used leftover coded material and patients are given the opportunity to object to their leftover material to be used in (clinical) research. Lung cancer samples were obtained from the PEMBRO-RT Phase 2 Trial [40] at the Netherlands Cancer Institute, which was approved by the institutional review board or independent ethics committee of the Netherlands Cancer Institute–Antoni van Leeuwenhoek Hospital, Amsterdam. All lung cancer patients provided written informed consent and consented to further analysis of patient material collected prior to and during the PEMBRO-RT trial. The research on prostate and bladder cancer samples was approved by the local Radboudumc Medical Ethical Committee (file number 2017-3934). All patients provided written informed consent to scientific use of archival tissue, unless deceased. The tonsil samples had been obtained anonymously from patients who had undergone routine tonsillectomies at Canisius Wilhelmina Hospital in Nijmegen, The Netherlands, and were processed in accordance with the regulations of the Dutch Committee on Regulation of Health Research.

### Simulation of artificial tissues

To generate synthetic *in silico* multiplex images, we used the cellular Potts model [41]. Briefly, cells are randomly placed in a 128^3^ µm^3^ volume and simulated at a resolution of 0.5^3^ µm^3^ per voxel, matching the resolution of real multiplex images. Cells consist of two compartments: a nucleus and a surrounding cytoplasm region. Cells were randomly assigned a phenotype, with associated nuclear and membrane-expressed markers matching real cells. The simulation operates by placing seed voxels of cytoplasm for each cell at a random location and letting them circularize for 25 Monte Carlo steps. Then, a nucleus seed voxel is placed inside each cell, and the simulation is run for a further 50 Monte Carlo steps so cells can settle into their final shape. Settings controlling the size of nucleus and cytoplasm per cell and adhesion strengths are described in Supplementary Table S1. The simulation temperature was set to T=20.

To simulate membrane-expressed markers, all voxels on the outer layer of a cell’s cytoplasm compartment (i.e. voxels having a neighbor that does not belong to the cell) are found and marked as a membrane. All voxels inside a cell’s nucleus are used for nuclear-expressed markers. An eight-voxel-thick slice is taken from the simulation volume, corresponding to a 4 µm tissue slice, matching our imaged tumor tissue slides. For each simulated marker, expression is simulated in either membrane or nuclear voxels, and signals are integrated along the viewing direction to construct an image. An exact cell segmentation mask is extracted from simulation data from the middle of the eight-voxel-thick slice.

### Human material

Tonsils (waster material) were collected from patients undergoing routine tonsillectomy at Canisius Wilhelmina Hospital in Nijmegen. Tonsils were stored at 4°C and processed within 24 h. The tonsils were cut in half, with one half was formalin-fixed and paraffin-embedded, and the other half was processed into a single-cell suspension. Fatty tissue was removed from the tonsil as much as possible with scalpels and placed into a gentleMACS C-tube (130-096-334, Miltenyi Biotec) with 5 ml RPMI containing 0.3 mg/ml Liberase (000000005401020001 Sigma) and 0.2 mg/ml DNAse I (18068-015, Thermo Fisher). Tonsil tissue was roughly cut into smaller fragments using scissors and was further dissociated into a single cell suspension on the gentleMACS (130-096-334, Miltenyi Biotec) program “Multi_C_01_01” two times with a 15 min incubation in between in a shaking water bath for 15 min at 37°C. 1×10^6^ cells were used for flow cytometry measurements and 1.5×10^7^ cells were fixed and embedded in paraffin with the AgarCyto cell-block preparation [42].

Melanoma specimens from patients treated at the Radboud University Medical Center (Radboudumc) were randomly included based on the availability of a resection specimen. Lung cancer samples were collected at the Netherlands Cancer Institute in the PEMBRO-RT Phase 2 Randomized Clinical Trial [40]. Bladder cancer samples were derived from patients who were treated for metastatic bladder cancer at the Radboudumc between 2016 and 2019. Archival tissue of both primary and metastatic tumor lesions was used. Prostate cancer samples are derived from patients who were treated in the Radboudumc and include archival tissue of both primary and metastatic tumor lesions.

Overall, we included 6 tonsil samples, 26 melanoma samples, 10 lung samples, 12 bladder samples, and 8 prostate samples.

### Flow cytometry

Single cells from tonsil ($10^{6}$) were stained with Fixable Viability Dye eFluor™ 780 (eBioscience, 65-0865-18, 1:1000) for 20 min at 4°C. After the wash step, cells were incubated with a mix of anti-CD3-BV421 (BD Bioscience, 563798, clone SK7, 1:25), anti-CD8-PerCp (BD Bioscience, 345774, clone SK1, 1:5), anti-CD45RO-APC (BD Bioscience, 340438, clone UCHL-1, 1:25), and anti-CD20-PE (Biolegend, 302306, clone 2H7, 1:10) for 30 min at 4°C. Next, cells were fixed, permeabilized with Foxp3/Transcription Factor Staining Buffer Set (eBioscience, 00-5523-00) and incubated with anti-FOXP3-alexa488 (eBioscience, 53-4776-42, clone PCH101, 1:8) for 30 minutes at RT. Flow Cytometry was conducted with the FACS Verse (BD Biosciences). Flow cytometry data was analyzed using FlowJo software (v10, Tree Star).

### Multiplex immunohistochemistry staining

Sections of 4 µm thickness (except for tonsil tissue, where we used 1 µm) were cut from FFPE tissue blocks containing tonsil, melanoma, and AgarCyto preparations, respectively. The slides were subjected to sequential staining cycles as described before [3], although now automated using Opal 7-color Automation mIHC Kit (NEL801001KT; PerkinElmer) on the BOND RX mIHC & ISH Research Platform (Leica Biosystems) as described previously [43]. All heat induced epitope retrievals were performed with Bond™ Epitope Retrieval 2 (AR9640, Leica Biosystems) for 20 min at 95°C. Blocking was performed with antibody diluent. Primary antibody incubations were performed for 1 h at RT. All secondary antibody incubations were performed for 30 min at RT. mIHC was performed with anti-CD45RO (Thermo Scientific, MS-112, clone UCHL-1, 1:3000) and Opal620, anti-CD8 (Dako, M7103, clone C8/144B, 1:1600) and Opal690, anti-CD20 (ThermoFisher, MS-340, clone L26, 1:600) and Opal570, anti-CD3 (ThermoFisher, RM-9107, clone RM-9107, 1:400) and Opal520, FOXP3 (eBioscience Affymetrix, 14-4777, clone 236A/E7, 1:300) and Opal540. For prostate cancer samples, anti-CD56 (Cell Marque, 156R-94, clone MRQ-42, 1:500) was used with Opal570 instead of anti-CD20. However, for this article, we ignored the CD56 marker by setting the corresponding channel to 0 before processing.

To visualize tumor cells, melanoma tissues were stained in the end with a melanoma mix consisting of anti-HMB-45 (Cell Marque, 282M-9, clone HMB-45, 1:600), anti-Mart-1 (Cell Marque, 281M-8, clone A103, 1:300), anti-Tyrosinase (Cell Marque, 344M-9, clone T311, 1:200) and anti-SOX-10 (Cell Marque, 383R-1, clone EP268, 1:5000) and Opal650 to visualize tumor tissue. Tonsil, bladder, and lung cancer tissues were finished with anti-pan cytokeratin (Abcam, ab86734, clone AE1/AE3 + 5D3, 1:1500) and Opal650 to visualize epithelial tissue. Finally, epithelial tissue in prostate cancer samples was visualized with a mix consisting of anti-pan cytokeratin (Abcam, ab86734, clone AE1/AE3 + 5D3, 1:1500), anti-EPCAM (Abcam, ab187372, clone VU-1D9, 1:1000) and anti-PSMA (Bio SB, BSB6349, clone EP192, 1:1000) and Opal650. Slides were counterstained with DAPI and mounted with Fluoromount-G (SouthernBiotech, 0100-01).

### Tissue imaging and data preparation

Slides were scanned using the PerkinElmer Vectra 3.0.4. Multispectral images were unmixed using spectral libraries built from images of single stained tissues for each reagent and unstained tissue using the inForm Advanced Image Analysis software (inForm 2.4.1, PerkinElmer). Per dataset, a selection of 1–2 representative original multispectral images per sample were used to train an inForm algorithm for tissue segmentation (into tumor/epithelium, stroma, background based on DAPI, tumor marker, and autofluorescence), cell segmentation, and phenotyping tool to recognize tumor cells, other cells, B cells and T cells populations (CD3^+^CD8^−^CD45RO^−^, CD3^+^CD8^−^CD45RO^+^, CD3^+^CD8^+^CD45RO^−^, CD3^+^CD8^+^CD45RO^+^, CD3^+^FOXP3^+^CD45RO^−^, CD3^+^FOXP3^+^CD45RO^+^). All settings applied to the training images were saved within an algorithm, and batch analysis of whole slides was performed. After checking the performance of each batch analysis, erroneous images were added to the inForm algorithms and retrained to improve the phenotyping of cells. Three iterations in total were performed per dataset to optimize the inForm algorithms. Component data files were used as input for ImmuNet.

### Artificial neural network

####  

The network was trained on patches of size 63×63×7 extracted from the annotated tiles so that the patches were centered at locations near the coordinates of the annotations. This allows us to use sparse point annotations to train the network. To smooth the loss function and make the network training more stable, we convert point annotations into proximity and phenotype maps (Supplementary Fig. S5). A proximity map represents closeness to the nearest cell center within a disk with a radius of 5 pixels (2.5 µm) drawn around an annotation, that is, its value equals 5 at coordinates of annotations and drops to 0 at the boundaries of disks drawn around them. For background annotations, we set the proximity map to −2. In the areas of unknown status (not covered by the disks centered at annotations), we set it to −1. A phenotype map is generated for each cellular marker in a similar way. However, it is initialized with 0 and, within a disk centered at an annotation, it is set to the corresponding Likert scale value mapped to a [0, 1] interval. Therefore, at the tile level, our labels are multichannel images constructed from the proximity and phenotype maps. Since most of the tile is not covered by annotations, we sample training patches only from locations of the proximity map with a known status (not equal to −1). One-eighth of such locations are sampled, with an upper bound of 1000 training patches per tile. Hence, one annotation is covered by multiple patches.

With this patch sampling strategy, a training patch label is a tensor of the values of the proximity and phenotype maps at the center of the patch; its size is 1×1×6. However, to encode information about the smoothness of the proximity and phenotype maps, we chose to use patches of size 3×3×6 as labels. A fully convolutional network architecture allows ImmuNet to predict the proximity and phenotype maps for an entire tile while being trained on such small patches. To detect and phenotype cells based on the network prediction, we first post-process the proximity pixel map prediction with a Laplacian-of-Gaussian filter as implemented in scikit-image [44], using parameters min_sigma = 3, max_sigma = 5, and threshold = 0.07. Then, for each detected cell location and each phenotype channel, we determine the mean value of that channel within a radius of 2 pixels around the center and use this as the pseudomarker expression value of the cell.

We used the Adam optimizer [45] during training with a learning rate of 0.001 and mean squared error (MSE) loss functions for both phenotype and distance pixel map predictions. Different weights were assigned to phenotype and distance map losses: 20 and 1 respectively. Here, we consider the Likert scale as a categorized representation of the annotator’s certainty about the marker’s positivity, continuous in reality. We also assume that the Likert scale is approximately evenly spaced. Under these assumptions, using the MSE as a phenotype loss function is appropriate since it reflects the underlying continuous nature of the annotator’s certainty in the training process. It also allowed us to both train on biologically gradual markers like CD45RO and include Likert scale annotations in training. When these assumptions are likely invalid, one should replace the MSE loss with categorical cross-entropy. We normalize each channel per tile by using the default percentile-based normalization from the CSBdeep Python library [46]. During training, we add Gaussian noise with a standard deviation of 0.1 to input and apply random data augmentations. Specifically, we perform horizontal and vertical flips and rotations and change the input image intensities randomly in the same way as StarDist [17] by first multiplying the input with a uniformly distributed scalar $s_{1}∼U(0.6,2)$ and then adding another uniformly distributed scalar $s_{2}∼U(-0.2,0.2)$. Convolutional layers perform batch normalization during training, except for layers in ResNet-like skip connections; fully connected layers perform dropout with a rate of 0.2.

The final network used in this article was trained on 183 678 patches of size 63×63×7 taken from 27 888 annotations. Training was run for 100 epochs, which took about 12 h. The neural network was constructed with TensorFlow [47], with important post-processing done in NumPy [48], SciPy [49] and scikit-image [44].

### Tumor and stroma tissue segmentation

Tumor and stroma tissue were identified using adaptive thresholding of different fluorescent stainings, as implemented by scikit-image [44]. For tumor segmentation, the tumor marker staining was thresholded; for stroma, a sum of all stainings was thresholded to first get the general tissue area, and the identified tumor area was subtracted to get all non-tumor tissue. Tissue-specific values were manually selected for tumor and stroma thresholds. Accurately segmenting tumor or stromal tissue is not trivial, but neural network methods based on U-net have shown success at tackling this task. While this simple segmentation algorithm likely does not achieve optimal performance, it is intended as *clear box* alternative to inForm software’s tissue segmentation. Replacing inForm with our own tissue segmentation in this manner allows us to present a tumor image analysis pipeline that is fully transparent and inspectable. Yet, our segmentation finds tumor and tissue in comparable regions to inForm software (Supplementary Fig. S4).

## Results

### Segmentation-based phenotyping fails in dense tissues even when segmentation is perfect

**Figure 1:**
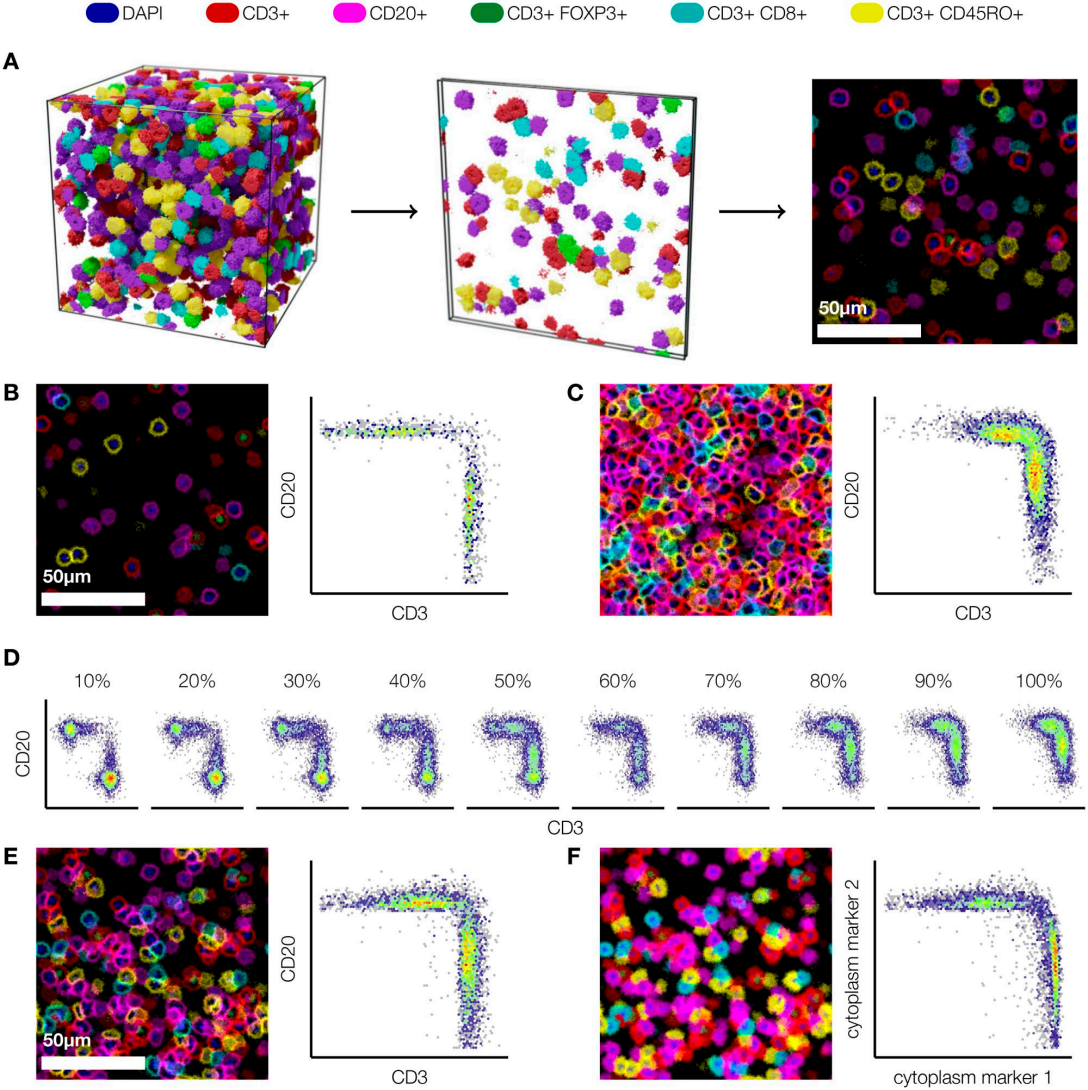
Segmentation-based phenotyping fails in dense simulated multiplex images. (**A**) To generate artificial immunohistochemistry images, we simulated cells at different densities within a 128^3^ µm^3^ volume, and cut 4 µm thick *in silico* slices spaced 8 µm apart. (**B, C**) Simulated tissue slices and corresponding scatterplots of CD3 and CD20 expression (membrane) on perfectly segmented cells at low (B) and high (C) cell densities. **(D)** At very high densities, individual cell populations are no longer identifiable (10% density: ∼3000 cells/mm^2^; 100% density: ∼30 000 cells/mm^2^). **(E, F)** Compared to membrane-expressed markers (E), markers expressed in the entire cytoplasm (F) are less affected by noise and spillover from adjacent cells

We asked to what extent the known difficulty of cell phenotyping in dense tissues is due to the technical artifacts affecting mIHC data, such as channel spillover and steric hindrance as opposed to the more fundamental problem that the segmentation task itself is ill-posed when we cannot assume that every pixel belongs to at most one cell. To address this question, we analyzed multispectral images generated by a computer simulation model. Unlike real images, such *in silico*-generated images have an available “ground truth”: we know exactly to which cell (or cells in case of overlap) each pixel in the image belongs. Therefore, we can use such images to reason about the hypothetical situation when we have a *perfect* segmentation algorithm available, which helps us to put an upper bound on the performance that any such approach can achieve. Specifically, to mimic real fluorescent histopathological images as closely as possible, we used the Cellular Potts modeling framework [41, 50–52]. We placed cells of realistic size (about 5–10µm in diameter) into a 3D space representing an unlabeled background structure, and “labeled” membrane, nucleus, or both, depending on the simulated cell type. We chose to simulate a panel that consists of DAPI, CD3, FOXP3, CD20, CD45RO, and CD8 cellular markers, which allows the identification of B cells (CD20+) and a few subtypes of a T cell (CD3+). We cut out thin slices (4 µm thickness) from these simulations for downstream analysis (Fig. 1A). We then simulated noisy expression of nuclear, cytoplasmic, and membrane markers on these cells and integrated the expression values along the z-axis to obtain simulated 2D multispectral images, which indeed had a striking similarity to real multispectral images (Fig. 1A and B); however, note that there is no spillover between channels or steric hindrance in these images.

We used these simulated tissue images to assess the performance of segmentation-based phenotyping when using membrane markers. To this end, we generated simple flow cytometry-like scatterplots of marker expression measured on segmented cells at varying densities, where we focused on only two membrane markers that represent CD3 and CD20 expression (Fig. 1B and C). As expected, this approach worked very well at low and medium cell densities (<80%; Fig. 1D): the plot clearly showed separate cell populations, which would be easy to classify in downstream analysis. However, at densities where cells overlapped, the separation between the different populations on the plots disappeared, creating the appearance of a single population with a continuum of expression of both markers. While it would still be possible to place arbitrary thresholds on these expression values to extract subpopulations, this approach would now risk either ignoring a substantial proportion of the cells, or misclassifying cells in the “double-positive” area. The problem was alleviated but not eliminated when we considered cytoplasm-based markers, which are less affected by cell overlap (Fig. 1E and F).

In summary, our simulation model shows that overlap between cells in dense tissues prevents simple threshold-based approaches to phenotyping from being successful. In real mIHC data, the problem is difficult enough that ML is needed to assign cell phenotypes reliably [19]; our simulations illustrate that this remains the case even with perfect segmentation. In other words, phenotyping in dense tissues is not a simple downstream task of segmentation. This insight motivated us to develop a multi-task ML pipeline to simultaneously localize and phenotype immune cells.

### A multi-task ML pipeline for simultaneous cell detection and phenotyping

**Figure 2:**
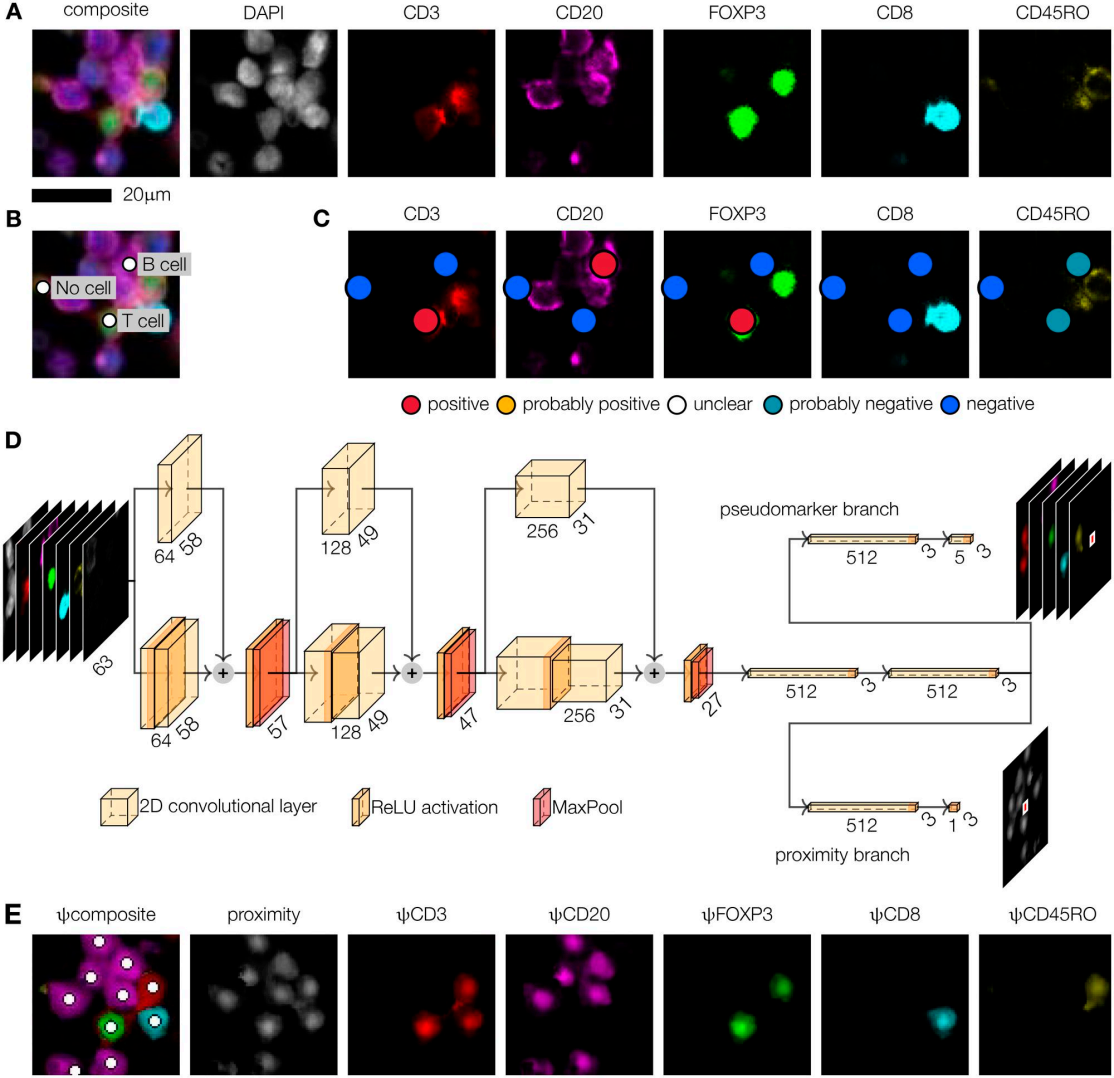
ImmuNet, an artificial neural network architecture for segmentation-free detection and phenotyping. (**A**) Multiplex immunohistochemistry imaging using a DAPI nuclear stain and a 5-marker panel designed to identify cytotoxic (CD8), regulatory (FOXP3), and memory (CD45RO) T cells (CD3) as well as B cells (CD20). A tumor marker was also added to the panel but was not used to determine immune cell phenotypes during annotation. (**B**) “Click” annotations of the locations of two cells and one background annotation (no cell). (**C**) “Decorations” of the annotations shown in (B) specifying the annotator’s certainty that each marker is expressed or not on the corresponding cell. (**D**) ImmuNet architecture consisting of nine convolutional layers arranged in three blocks with ResNet-like skip connections, followed by a convolutional layer to reduce feature map size to 3 × 3, a fully connected layer, and two output branches of two fully connected layers each. The network is trained on 63 × 63 × 7 input patches (DAPI, the five immune cell markers, and the tumor marker) and generates 3 × 3 output matrices containing the proximity to the nearest cell (proximity branch) and the expression of each marker on the nearest cell (pseudomarker branch). (**E**) Output of the ImmuNet network on the input shown in (A). White circles show cell positions detected by Laplacian of Gaussian post-processing of the proximity map

We developed our pipeline for mIHC data of formalin-fixed paraffin-embedded (FFPE) tissue. Specifically, our mIHC method uses the Opal tyramide signal amplification technique and multispectral imaging [53]. When used in conjunction with the Vectra 3 system or its successor, the Polaris, this method can combine six markers or more within one FFPE tissue section. However, because of the serial staining protocol, panels have to be optimized carefully [3]. Using this technique, we developed a seven-color lymphocyte panel to detect different lymphocyte populations within tissue consisting of CD3, FOXP3, CD8, CD45RO, CD20, a tumor marker (such as pan-cytokeratin or a melanoma-specific antibody cocktail), and DAPI (Fig. 2A).

In previous studies, we used the software inForm (PerkinElmer, v. 2.4.10) in conjunction with in-house developed downstream quality control and analysis software to segment and phenotype cells in mIHC images [3, 29, 30, 43, 54–56]. Given our familiarity with this software, its relatively widespread use in the research community, and our experience in fine-tuning it to specific tissues, we use it as a baseline method for comparison throughout this article. The inForm software uses an ML algorithm to assign every pixel in the image to at most one cell and subdivides each cell into compartments such as “nucleus” and “membrane”. Subsequently, it extracts marker expression information for each channel (e.g. mean expression, range of expression, variance of expression) along with morphological features such as size and shape indices. Users can manually annotate cells with known phenotypes to train a multinomial logistic regression classifier model that assigns a phenotype to each cell [57]. This approach can be expected to work well for isolated cells, but as shown earlier, is conceptually challenging when cells overlap in dense tissues. Unfortunately, especially immune cells are often found in densely packed structures such as secondary lymphoid organs or tertiary lymphoid structures, and immune infiltrates in tumors, which are also often dense.

As is common in ML, we started by formulating our task in terms of the desired input and output. Existing neural network architectures for cell segmentation are typically based on images containing manually drawn cell outlines. Generating such cell boundary annotations is a time-consuming task. While sparse tissues are easier to annotate, networks trained on such images may perform poorly on dense structures that they did not see during training. For these reasons, we formulated two key requirements for our design: (i) users should only have to annotate the location of each cell (click annotation) instead of its entire shape (polygon annotation), given that we do not intend to use the shapes anyway; (ii) users should not have to annotate training images fully, because even for a human expert it is difficult to identify every cell in dense tissues. We developed a custom annotation tool that allows users to place annotations simply by clicking on the center of a cell of interest (Fig. 2B). In a second step, which we call “decoration”, users can verify and fine-tune the locations of the annotated cells and rank the expression of each phenotyping marker on a five-point Likert scale (Fig. 2C). Importantly, the Likert scale represents the user’s certainty that a cell does or does not express a particular marker rather than a qualitative judgment on expression intensity. This allows annotators to specify that they are uncertain about some cases, which can then be resolved by discussing these cases with a larger team of annotators and getting input from experts. In our case, such discussions took place only for a small number (tens) of cases.

We then designed an artificial neural network (ANN) architecture that processes the location and phenotype annotations to generate two types of output per pixel: (1) the proximity of this pixel to the nearest center of a cell; (2) the expression of each phenotyping marker on the nearest cell (Fig. 2D and E, Supplementary Table S2). The network has a fully convolutional structure (Fig. 2D, Supplementary Table S2) that allows it to generate whole-image predictions during inference despite predicting only small patches of output during training (we use a patch size of 3 × 3 pixels to encode at least some information on the smoothness of the proximity function). This setup makes it straightforward to process sparsely annotated data: only pixels near annotated cells are considered during training. To be able to distinguish background and foreground, we allow users to place special annotations in regions that do not contain any cell of interest. Our ANN architecture incorporates elements from the DeepCell network [12] and a key idea of Wang *et al*. [58], who trained a network on a similar distance transformation of cell locations.

Hence, our ANN architecture, which we called ImmuNet, generates maps that encode information about cell location and phenotypes, but not cell shape. These maps can be processed using any object detection algorithm. We found a simple Laplacian of Gaussian (LoG) blob detection algorithm to work well for our purposes (Fig. 2E). The final output is a list of spatial coordinates of each detected cell and its expression of each marker quantified by what we call “pseudomarkers” (ψ). This kind of data is familiar to many biologists as it closely resembles the output of flow cytometers but with added spatial information. Indeed, we found that converting ImmuNet data to the flow cytometry standard (FCS) format was an effective way to allow users to explore their multi-dimensional mIHC data using familiar software.

### Training and evaluation of ImmuNet on different types of tissues

**Figure 3:**
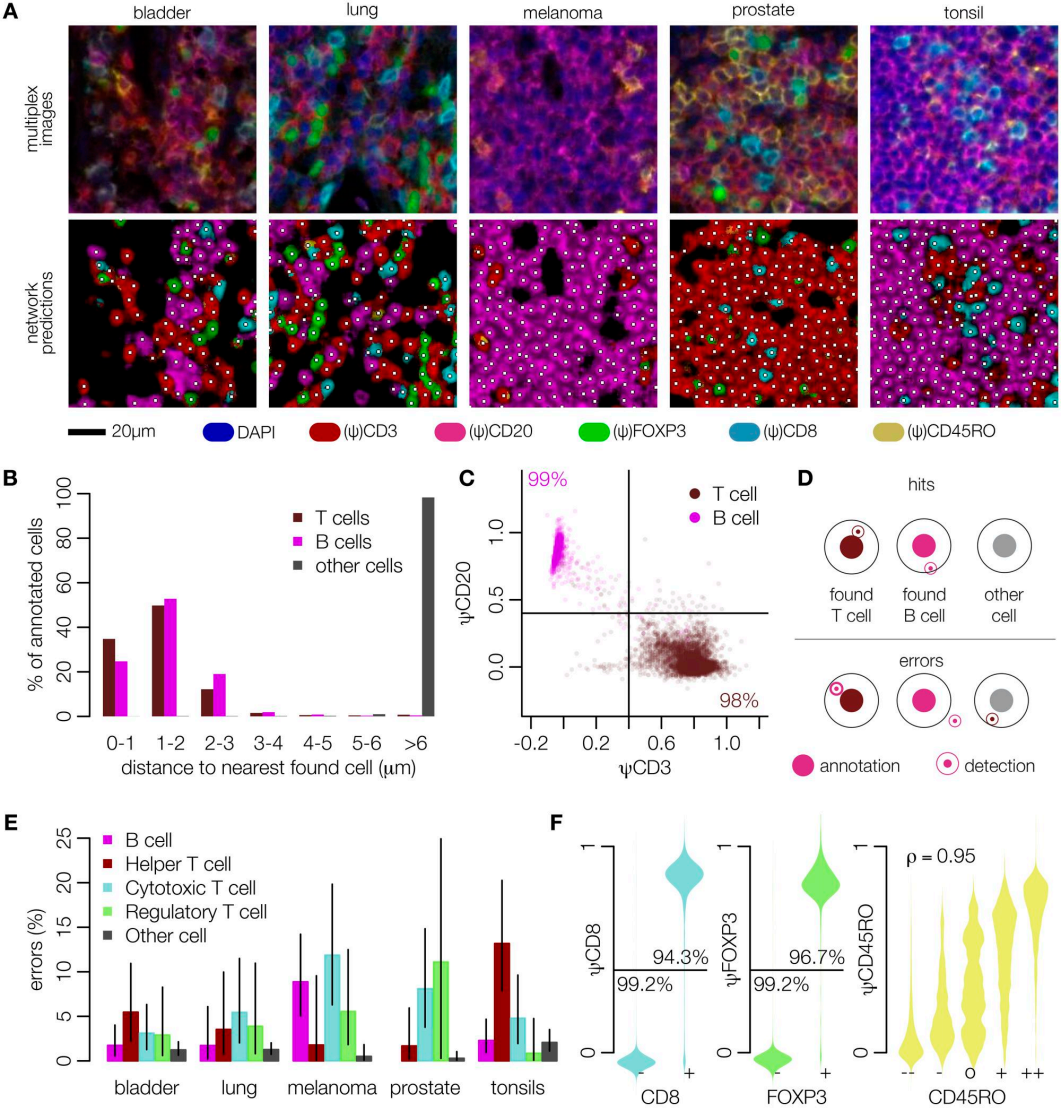
Identifying and phenotyping B- and T cells using ImmuNet. (**A**) Representative input images from four different types of tumor samples and a tonsillitis sample, and the corresponding ImmuNet predictions, focusing on dense lymphocyte clusters. (**B**) Distribution of distances between annotated cells and cells identified by ImmuNet. (**C**) Expression of the CD3 and CD20 pseudomarkers (ψ) on detected cells. (**D**) Definition of correct and faulty detections used in (**E**), which shows the error rates of ImmuNet per tissue type compared to our baseline method. The detection radius used is 3.5 µm. Error bars: 95% confidence intervals (Clopper–Pearson method). The wide confidence interval for regulatory T cells in prostate is a result of the low number of such cells found in prostate samples. (**F**) Distribution of pseudomarkers on detected cells compared to annotated marker expression on the nearest annotated cell. For CD8 and FOXP3, we grouped weakly and strongly positive or negative annotations

Having defined our network architecture, we proceeded to collect data for annotation, training, hyperparameter tuning, and evaluation. To this end, we created a database consisting of whole-slide mIHC images from four different types of human tumor samples (bladder cancer, lung cancer, melanoma, and prostate cancer; see section Materials and methods) as well as tonsil material from tonsillectomies (Fig. 3A). All samples were stained using our T cell panel mentioned above except for the prostate samples, where we did not use the CD20 channel.

Using our custom-built browser-based tools, we annotated and decorated thousands of lymphocytes of various types. In addition, we used “background” annotations, which include tumor cells, other cells—those that do not express any of the lymphocyte markers, which could be tumor cells, stroma cells, or structures that are not cells but look similar—and sites without a cell to provide the model with negative examples. To enrich our set of annotations for difficult cases, we performed several rounds of model training. At the end of each round, the output of the network was visualized and manually inspected to find areas where the network was making mistakes. Then, additional annotations were made at the problematic sites and the model was retrained. We stopped when we had accumulated 34 458 cell annotations, at which point we no longer found obvious problems with our network by visual inspection. We also trained baseline inForm segmentation and phenotyping algorithms on the same data, which, unlike the ImmuNet approach, required training a dedicated algorithm for each tissue type and sometimes multiple algorithms per tissue type if there were substantial differences between batches (such as changed microscope configuration settings).

Since our multispectral images consisted of stitched individual tiles (1332 × 996 pixels), we separated the annotated tiles into training and validation sets to evaluate generalization to unseen tiles. Specifically, we used 20% of the annotations for validation and balanced the distribution of tissue types and phenotypes between training and validation data. The final training set comprised 27 888 annotations (lymphocytes and background annotations), leaving 6570 annotations for our initial validation; additional validation steps were also performed, and are described later in this article. The choice to split our data by tile, rather than by slide, was made to make it easier to obtain both training and validation sets that represent all cell and tissue types, given the large heterogeneity between slides.

The training data were used to tune LoG parameters and to define hyperparameters as follows: after training our network and setting the LoG parameters, we measured the distance to the nearest detected cell for each annotated cell (Fig. 3B). For the vast majority of annotated T- and B cells, ImmuNet detected a lymphocyte no further than 3 µm away, which was rarely the case for non-lymphocyte annotations such as tumor cells or stromal cells. The expression of the CD3 and CD20 pseudomarkers closely matched the annotated phenotype: for 98% of the annotated T cells and 99% of the B cells, the closest detected cell expressed the corresponding pseudomarker—but not the other pseudomarker—at an intensity of 0.4 or higher (Fig. 3C). Thus, in the training data, most of the annotated cells were correctly detected by the ImmuNet pipeline when using a distance cutoff of 3.5 µm and an intensity cutoff of 0.4.

We next determined the pipeline’s performance in the detection of T cell phenotypes. While making annotations, we generally found it easy to decide on positivity for CD20, CD3, CD8, and FOXP3 markers. We therefore binarized the Likert scale annotations to “positive” and “negative” for these markers and discarded the few annotations for which this distinction could not be made. The status of the CD45RO marker—which in principle can be expressed on most T cells—was more difficult to assess, so we kept the original Likert values. Likewise, we used the established cutoff of 0.4 to binarize the predicted pseudomarker expression values except for ψCD45RO. In this way, we defined five different phenotypes as a basis for evaluation: B cells (CD20^+^CD3^-^CD8^-^FOXP3^-^), helper T cells (“Th” for short; CD3^+^CD8^-^FOXP3^-^CD20^-^), cytotoxic T lymphocytes (“CTL”; CD3^+^CD8^+^FOXP3^-^CD20^-^), regulatory T cells (“Treg”; CD3^+^FOXP3^+^CD8^-^CD20^-^), and other cells (CD20^-^CD3^-^CD8^-^FOXP3^-^). We treated any other combination of predicted CD20, CD3, CD8, and FOXP3 levels as an invalid prediction. We then evaluated the entire cell detection and phenotyping process as follows: for each annotated B- or T cell, we require the closest detected lymphocyte to be no further than 3.5 µm away, and it must have the same phenotype. For other annotations, no B- or T cell must be detected by the network within a 3.5 µm radius (Fig. 3D).

Using these definitions, we found that the ImmuNet error rate in the validation data was below 10% for B cells and for most T cell subtypes (21 out of 24 categories tested; Fig. 3E), and below 5% for 16 out of the 24 categories tested. False positives (i.e. “other cell” annotations where the network predicted a lymphocyte in close proximity) were also acceptably low with tonsils showing the largest false-positive rate at only 2.1%. For every possible combination of marker and tissue, comparing these values to the error rates of our baseline inForm algorithms (which were also trained on cell annotations collected from these datasets) showed that ImmuNet significantly outperforms inForm in lymphocyte detection and phenotyping: error rates measured on the same validation dataset were above 20% for B cells and above 10% for T cell subtypes across all tissue types, sometimes reaching values as high as 40% even after re-training each inForm algorithm twice to optimize performance (section Materials and methods; Supplementary Fig. S1).

Analyzing the pseudomarker values in more detail for T cell subsets likewise showed excellent agreement between annotated and predicted expression (Fig. 3F, left). Since the annotations of the CD45RO marker were more uncertain, we visualized pseudomarker distributions for each value of the Likert scale (Fig. 3F, right), which confirmed that it would not be appropriate to use a binary cutoff for this marker, as too much information would be lost. Instead, we characterized the agreement between annotations and predictions using the polyserial correlation, which was high (0.95). Importantly, when an annotator was more certain (first and last levels of the Likert scale), the pipeline’s prediction matched the annotator’s judgment in most cases.

Taken together, these results showed that ImmuNet can detect and phenotype B cells, helper T cells, cytotoxic T cells, and regulatory T cells using a pseudomarker expression cutoff of 0.4, whereas CD45RO expression needs to be evaluated on a continuum, avoiding binarization. Error rates were consistently below 10% and often below 5%, a major improvement compared to our previously used baseline method.

### Validation on fully annotated regions of interest

**Figure 4:**
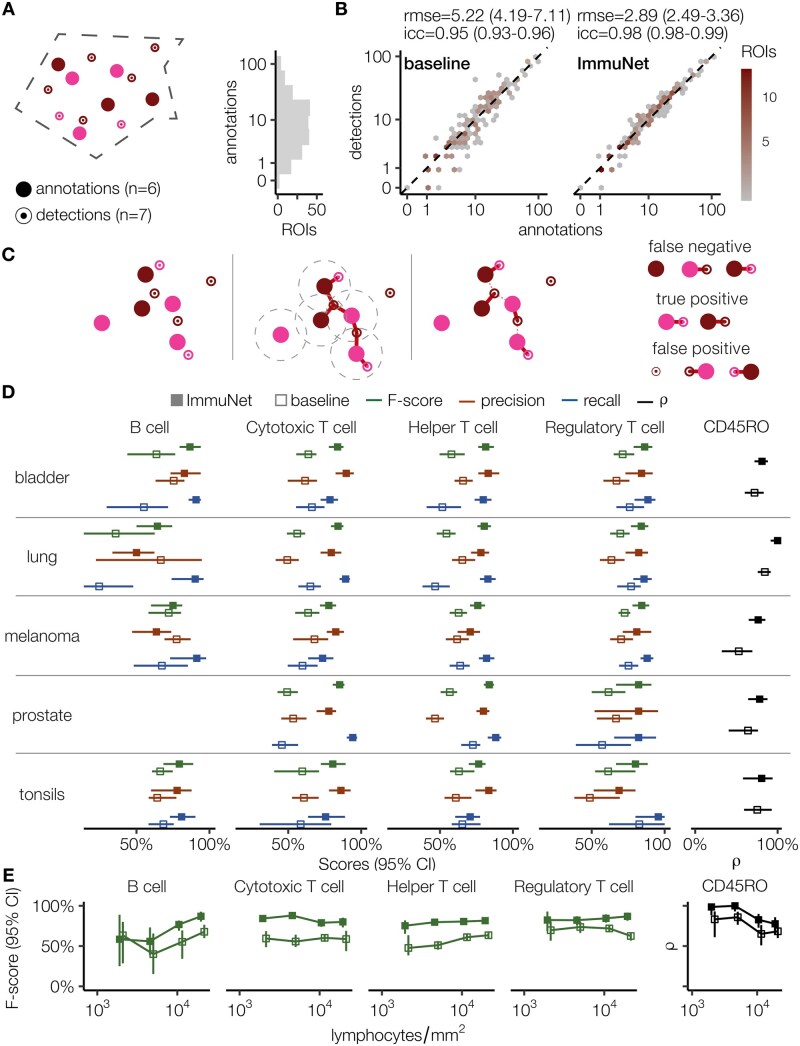
Validation of ImmuNet lymphocyte detection across tissue environments, phenotypes, and densities. (**A**) Sketch of an ROI with human annotations and detections made by ImmuNet, with different colors representing different phenotypes; the histogram shows the distribution of the number of annotations per ROI. (**B**) Number of annotations and detections for the baseline algorithm and ImmuNet, visualized using hexagonal binning. 95% confidence intervals of RMSE are computed with bootstrap. (**C**) Illustration of how we define TPs, FPs, and FNs based on optimal pairwise matching of annotations to detections. (**D**) ImmuNet and inForm performance across tissue types and cell phenotypes. (**E**) ImmuNet and inForm F-scores across density profiles and cell phenotypes. Error bars in (D, E): bootstrapped 95% confidence intervals

Encouraged by the low error rates found in this initial analysis, we performed a second round of testing designed to better identify additional potential issues. First, even low false-positive rates might still be problematic if entire slides are analyzed that contain almost no lymphocytes: in such cases, the majority of detected lymphocytes could be errors. Second, we noticed that hypersegmentation of cells—where a single cell is being detected as multiple cells—was a common problem with our inForm algorithms and was not being picked up in our initial test due to our focus on single cells. Additionally, when making sparse annotations, annotators might be biased towards the selection of easier cases. Therefore, we next fully annotated 193 regions of interest (ROIs; Fig. 4A) representing different tissues, cell compositions, and density levels.

First, we determined the agreement between the number of annotated and detected cells in each ROI by calculating the intraclass correlation coefficient (ICC)—a measure of interrater agreement that ranges from -1 (perfect disagreement) to 1 (perfect agreement)—and the root mean square error (RMSE) between these values for ImmuNet and the inForm baseline (Fig. 4B). While inForm achieved an ICC of 0.95 and an RMSE of 5.22, ImmuNet improved both metrics, increasing the ICC to 0.98 and reducing the RMSE to 2.89 cells. Figure 4B highlights that inForm tends to miss lymphocytes frequently and that this may be a bigger issue on average than hypersegmentation. In contrast, ImmuNet seems to over- and underestimate the number of lymphocytes similarly often.

Next, we assessed the accuracy of cell phenotyping and localization within ROIs. For B cells, helper T cells, cytotoxic T cells, and regulatory T cells, we used precision, recall, and F-score as evaluation metrics, whereas the accuracy of the predicted CD45RO expression was evaluated by measuring the association of Likert scale annotation with predicted positivity. We used polyserial correlation for ImmuNet and polychoric correlation for inForm, since inForm performed a binary CD45RO classification. To compute these metrics, we needed to match detections and annotations in each ROI to each other. For this, we used a standard maximum matching algorithm [59], which matches as many cells as possible while respecting the distance cutoff of 3.5 µm. This matching allows us to calculate the number of true positives (TPs), false positives (FPs), and false negatives (FNs) in each ROI, as illustrated in Fig. 4C: we consider the predicted cell to be a TP if it is matched with an annotation of the same phenotype. An FP is defined as a detection that is unmatched or matched to an annotation with a different phenotype. Conversely, unmatched annotations and annotations matched with a prediction of a different phenotype are considered FNs. For the CD45RO marker, the polyserial or polychoric correlation between the Likert annotation and the pseudomarker expression was computed for the matched CD3^+^ annotations and detections.

We performed this evaluation across different tissue types (Fig. 4D) and density profiles (Fig. 4E, Supplementary Fig. S2). Density profiles were obtained by binning the log-scaled range of annotated lymphocyte densities in all ROIs. F-scores, precision, and recall for phenotypes and tissue environments (density profiles) are calculated by bootstrapping upon aggregating TPs, FPs, and FNs across all ROIs. Polyserial (polychoric) correlation for the CD45RO marker is calculated in the same way. These results demonstrate that ImmuNet outperforms inForm across lymphocyte phenotypes, different tissue types, and density profiles. Interestingly, recall generally contributed more to the performance improvement brought by ImmuNet than precision. Particularly, when the difference in F-scores was large, it was most often due to the difference in recall (B cells and helper T cells in bladder and lung; cytotoxic T cells in prostate). This is further evidence that inForm is more prone to missing cells than to false detections. However, there were a couple of cases of a dramatic increase in precision (cytotoxic T cells in lung and tonsils; helper T cells in prostate). Importantly, ImmuNet performance did not drop much at higher tissue densities, except for the prediction of CD45RO expression. In contrast, we did notice an interesting drop in inForm performance for regulatory T cells at high densities, mainly due to decreased precision (Supplementary Fig. S2). Since FOXP3 is a nuclear marker, its measurements should still be fairly reliable even at high densities. However, it does rely on a reasonable initial segmentation, since regulatory T cells are rare and often surrounded by cells of other types.

Taken together, our analyses on two differently designed validation datasets demonstrated the robust performance of ImmuNet across tissue types and cell densities and that it significantly outperformed the baseline method “inForm”.

### External validation of ImmuNet results using flow cytometry measurements of the same tissue

**Figure 5:**
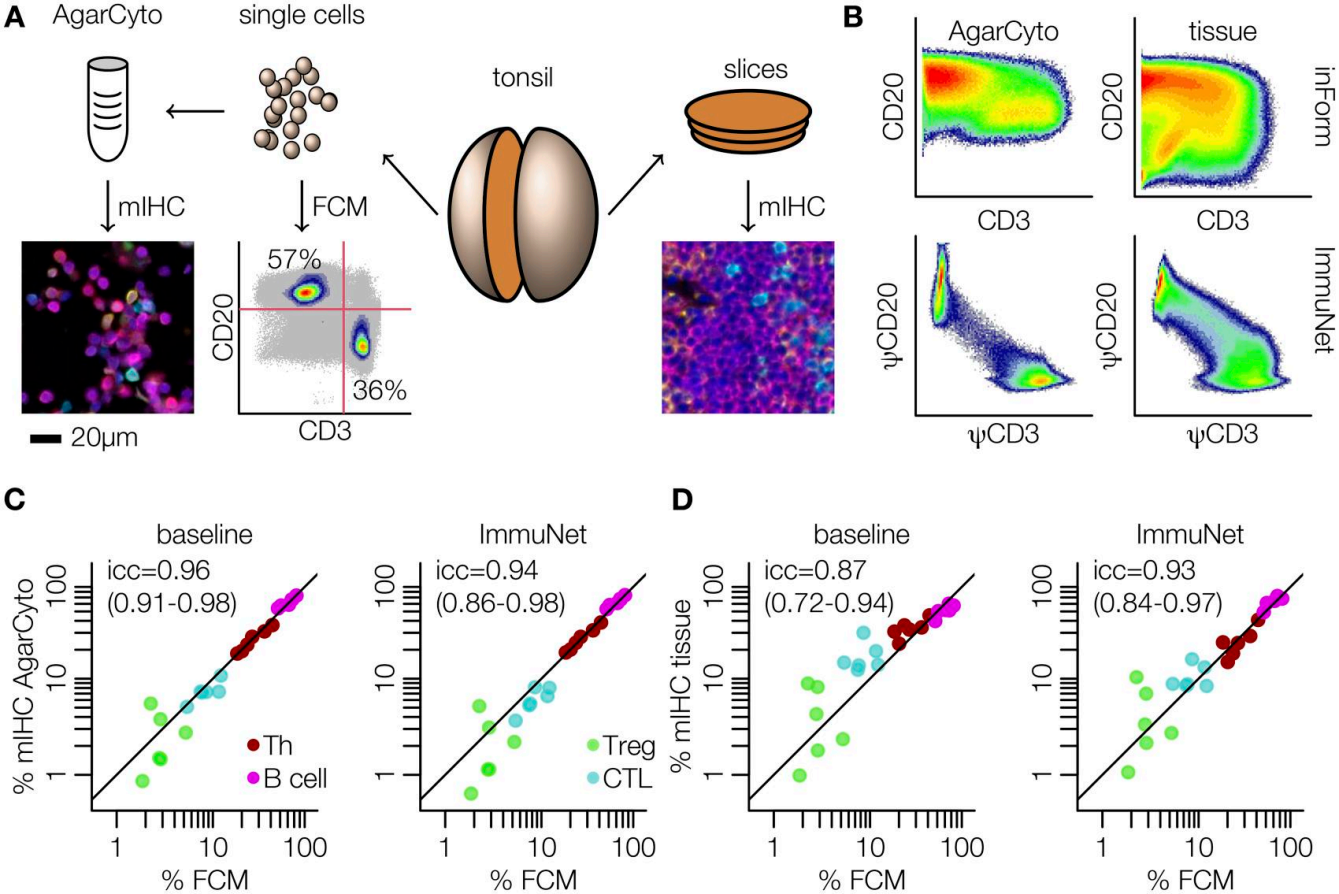
External validation of ImmuNet-derived phenotype abundances. (**A**) Tonsils were cut in half with one half sliced and imaged by mIHC, and the other half dissolved and further processed by flow cytometry or an AgarCyto preparation, which was also imaged by mIHC. **(B)** Directly measured expression of CD3 and CD20 on cells segmented from the mIHC images by inForm compared to the expression of the corresponding pseudomarkers on ImmuNet-detected cells. **(C, D)** Cells in AgarCyto (C) or direct FFPE (D) mIHC images were either phenotyped using the multiparametric classifier implemented in inForm (baseline), or using a threshold of 0.4 on the ImmuNet pseudomarkers. Concordance to flow cytometry measurements (FCM) from the same tonsils is shown and quantified using the intraclass correlation coefficient (icc) and its 95% confidence interval

Up to this point, our performance evaluations were based on manually annotated cells. While this is a standard procedure in ML, we noticed that human annotators did not always agree in their judgments about cell counts and phenotypes; upon closer examination of prediction errors, we repeatedly found cases where the ground truth was in fact debatable. We therefore designed an experiment to compare our mIHC phenotyping data to external reference measurements rather than human annotations. Flow cytometry is a mature and widely used non-spatial method for cell phenotyping. Because cells are dissociated and imaged one by one in a flow cytometer (rare duplicates can be filtered out in post-processing), and the entire exterior of a cell is accessible to the cytometer, marker expression can be measured more reliably compared to mIHC imaging. We therefore decided to use flow cytometry as an external control for the relative lymphocyte phenotype abundances estimated by mIHC-based phenotyping. To this end, we obtained fresh human tonsil tissue. Tonsils are a useful test case for our analysis because they contain extremely densely packed B- and T cell areas that are notoriously difficult to process for segmentation algorithms. For further processing, we split each tonsil in half (Fig. 5A). One half of each tonsil was dissociated into single cells and analyzed by both flow cytometry and an FFPE AgarCyto cell block preparation [42] subjected to mIHC. The other half was directly FFPE and subjected to mIHC. We then quantified the number of B cells, T helper cells, cytotoxic T cells, and regulatory T cells as a proportion of all B- and T cells for each of the three preparations. Our motivation to include mIHC images of AgarCyto preparations in this analysis was that cells in such preparation are generally isolated. Therefore, if our baseline method achieves acceptable performance on these data, it would support our hypothesis that high cell density is a major source of errors in segmentation-based phenotyping algorithms.

Scatterplots of CD3 versus CD20 expression clearly showed distinct peaks representing T- and B cells for the flow cytometry data (Fig. 5A). While two separate peaks were still somewhat apparent from the AgarCyto preparation analyzed with the inForm baseline method, these disappeared when directly imaging the dense tonsil tissue (Fig. 5B), resembling our initial findings on simulated data (Fig. 1B). In contrast, separate peaks in the expression of the ImmuNet pseudomarkers were still readily identifiable for both the AgarCyto preparation and direct FFPE tissue imaging (Fig. 5B). To phenotype the cells based on their expression profiles, we again used the positivity threshold of 0.4 identified previously for CD3, CD8, FOXP3, and CD20 (Fig. 3). Given that similar direct thresholding of expression markers would not seem sensible for the inForm data (Fig. 5B), we instead trained and applied the inForm phenotyping classifier, which can take many additional features into account, to determine the baseline performance.

Reassuringly, our analysis showed good agreement between AgarCyto-mIHC and flow cytometry measurements (FCM) in both the baseline analysis and the ImmuNet data (Fig. 5C). On the FFPE mIHC images, the agreement of the baseline method with flow cytometry data degraded somewhat from ICC = 0.96 to ICC = 0.87, mainly due to an apparent overcounting of CTLs, while the ImmuNet-based agreement remained very similar (ICC = 0.94 versus ICC = 0.93). We noticed that these agreement measurements were strongly influenced by the Treg population, which was hard to gate and, therefore, less reliable on the flow cytometry data (Supplementary Fig. S3A). We therefore performed the same analysis without the Treg population, which gave similar results for ImmuNet but a more marked loss in performance for the baseline method on the tissue images (Supplementary Fig. S3B). As mentioned previously, this drop in performance illustrates the difficulties faced by segmentation-based algorithms in dense tissues. However, an important caveat of this analysis is that one does not necessarily expect perfect agreement between flow cytometry and tissue images because suspension could lead to the partial loss of some cell populations.

In summary, our comparison of mIHC phenotyping with flow cytometry analysis of the same tissue showed generally good agreement between the two methods, provided that cells were easy to “gate” in both methods. Our analysis suggested that immune cell phenotyping achieved by ImmuNet was at least on par with that achieved by our segmentation-based baseline method, which appeared to struggle with the oversegmentation of CTLs in the dense tonsil tissue. This result appears to contradict our earlier evaluation on fully annotated ROIs, in which low recall appeared to be the larger issue for our baseline method; however, note that our previous analysis also required the cells to be in the correct position, whereas here we compared only the counts. Together, these analyses therefore reveal issues with both precision *and* recall in our segmentation-based baseline method.

### Spatial analysis of tumor microenvironments reveals distinct infiltration patterns and local interactions of lymphocytes

To illustrate the applicability of the ImmuNet approach in cancer research, we performed a spatially resolved analysis of tumor-infiltrating lymphocytes (TILs) in the tumor microenvironment. Such analyses are common in the literature because TIL density has prognostic or predictive value in different types of cancer, including melanoma [28, 60], lung cancer [61], and colorectal cancer [62]. Based on TIL density within and around the tumor, tissue samples are often described as “hot” (TIL-rich), “excluded” (TIL-rich in stroma, but not tumor), and “ignored” (TIL-poor) [63], or TIL distribution is examined using more refined measures of effective infiltration [29, 64]. Multiplex imaging techniques add phenotype information to such approaches, allowing the analysis of interactions between different types of cells [23].

**Figure 6:**
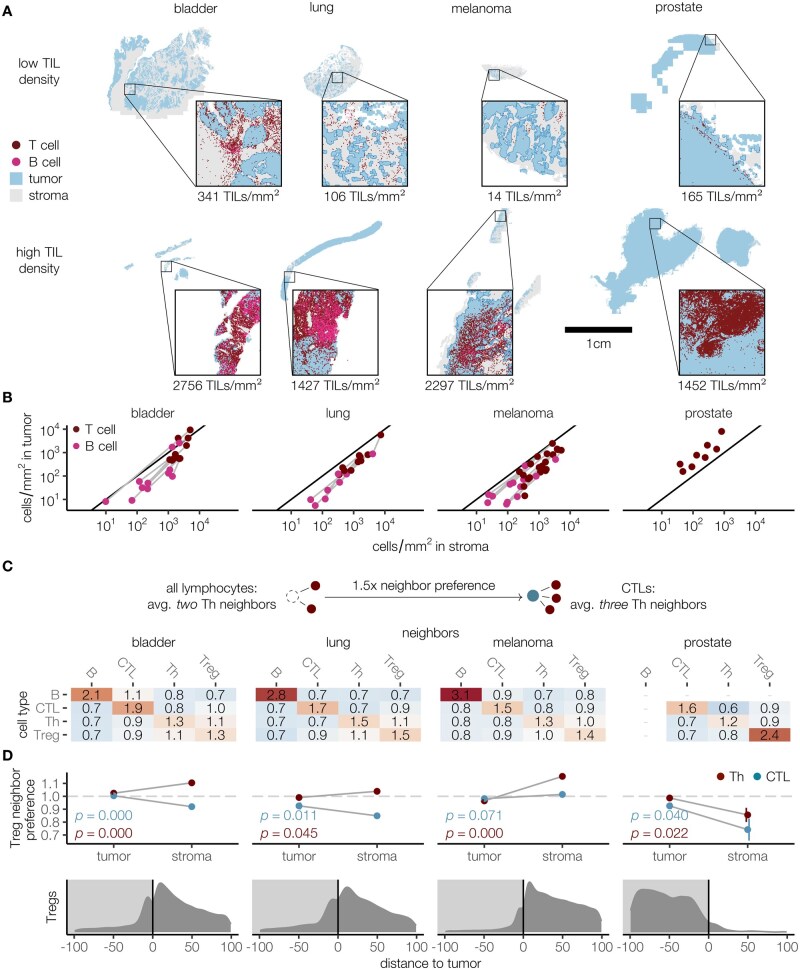
Quantifying immune cell infiltration and cell-cell interactions in the tumor microenvironment. (**A**) Representative sections in different tissue environments with low and high TIL density. (**B**) Density of B and T cells inside or outside the tumor (100 µm from the border); we could not quantify B cells in prostate sections since they were not stained with CD20. (**C**) Analysis of neighboring cells at up to 14 µm between cell centers. The average occurrence of different cell types as neighbors is calculated for neighbors of lymphocytes in general and for neighbors of specific cell types. The ratio between these numbers is deemed as the preference of a certain cell type to have another cell type as a neighbor. The matrices show these numbers for each combination of cell types (rows: reference cell type; columns: neighboring cell type). (**D**) Stratified analysis of Treg neighbor preference by tissue type (top) and frequency of Tregs by distance to stroma/tumor border (negative numbers are inside the tumor). *P*-values from a T-test of the null hypothesis of no difference between tumor and stroma areas are shown. Bladder: *N* = 11 whole-slide images; lung: *N* = 10; melanoma: *N* = 23; prostate: *N* = 8

We used ImmuNet to analyze TILs in our whole-slide mIHC images of bladder cancer, lung cancer, melanoma, and prostate cancer tissue specimens (Fig. 6A), applying a simple threshold-based segmentation algorithm to distinguish tumor and stroma (section Materials and methods; Supplementary Fig. S4). Similar to a previous study [64], we measured the density of T cells (combining all phenotypes) and B cells in the invasive margin, defined as a region within 100 µm around the tumor-stroma boundary. For bladder cancer, lung cancer, and melanoma, the density of TILs in stroma was consistently higher than or comparable to their density in tumor (Fig. 6B). In the prostate cancer samples, there was a different picture where the density of cells outside the tumor was consistently lower.

Next, we assessed local cell-cell interactions between different lymphocytes on a smaller spatial scale. We designed a “neighbor preference” metric to quantify which cell types preferentially appear next to each other. The metric is calculated by determining how often a certain type of cell is present as a neighbor of other lymphocytes in general (e.g. helper T cells as neighbors of all lymphocytes) and how often they are neighbors of a specific cell type (e.g. helper T cells as neighbors of cytotoxic T cells specifically; Fig. 6C). The ratio between these counts indicates how frequently two cells of the investigated types appear next to each other compared to a random assignment of cell types. This analysis revealed similar interaction patterns across the different tissue types (Fig. 6C): all cell types co-localized with cells of their own type (neighbor preference >1), whereas cells of different types were less likely to be close neighbors (neighbor preference <1). B cells had overall the greatest tendency to co-localize with their own kind, which could be due to the presence of follicle-like structures in the tumor microenvironment.

As regulatory T cells showed the weakest overall preference for or against other T cell phenotypes (as seen from the Th-Treg and Th-CTL values being close to 1), we stratified this interaction further by distinguishing between intra- and peritumoral cells (Fig. 6D, top row). In all tissues, intratumoral regulatory T cells had no strong preference for helper T cells or cytotoxic T cells. In the stroma, however, they preferred helper T cell neighbors over cytotoxic T cells. For prostate cancer, we observed an apparent preference for homogeneous contacts, but this is likely a consequence of the very low number of peritumoral regulatory T cells in these samples (Fig. 6D, bottom row).

In summary, the ImmuNet pipeline allowed us to quantify infiltration patterns in different environments, determine cell-cell interactions, and stratify cell-cell interaction patterns by spatial compartment. This illustrates that cell segmentation is not always necessary to conduct spatial analyses of immune landscapes in tissues.

## Discussion

We have developed, implemented, trained, and tested ImmuNet, an ML pipeline for simultaneous and segmentation-free localization and phenotyping of immune cells in mIHC imaging. Although relatively little information is used to train ImmuNet compared to segmentation-based ML pipelines such as StarDist [17], Cellpose [16], and Mesmer [15], we found it to perform well across diverse types of tissues, including very challenging dense environments. By design, ImmuNet is particularly well suited for applications where the cell shapes are not required for downstream analysis. This should often be the case for immune cells because lymphocytes tend to lose their physiological shape in dead tissue and round up, leaving little meaningful variation. Our example analysis shows that quite sophisticated spatial information can be extracted even without cell shapes. However, when cell morphology is genuinely important for the research question at hand, one could still combine ImmuNet with cell segmentation of the same tissue. Indeed, the pseudo-marker profiles generated by the ImmuNet network could simply be added to the image as additional channels for further analysis in segmentation software such as CellProfiler [65] or ilastik [66]. Alternatively, a cell segmentation network like Mesmer [15] could be extended with branches to generate ImmuNet pseudomarkers alongside segmentation maps, or the locations of the cells detected by ImmuNet could be used as prompts for foundation models such as the Segment Anything Model [67]. Thanks to its fully convolutional architecture, ImmuNet’s inference time does not depend on the cell density in an image. Importantly, we validated ImmuNet in a research setting, in which we are able to perform manual quality controls and re-train the network if any batch effects are detected. Generalization to unseen slides and tissues, and robustness to batch effects, should still be investigated more carefully.

Even though there are many existing ML methods for cell segmentation and phenotyping in tissue images, we are not aware of other multi-task architectures that combine cell localization and phenotyping in the way that ImmuNet does. To our knowledge, the most similar existing approach is the one proposed by Shui *et al.* for single-stained (PD-L1) IHC data [38]. Their approach differs from ours by restricting the possible positions of the cell center to pre-defined anchor points, which are matched to ground-truth annotations in a manner similar to our matching between network predictions and annotations. Phenotyping is treated as a classification problem. For future work, it would be interesting to generalize their approach to multiplex data (e.g. by performing the classification task channel by channel) and compare the performance to ImmuNet.

While the imaging data in this paper was exclusively based on our T cell panel, the procedure works in the same way for other panels, such as our checkpoint molecule expression panel [3] or dendritic cell panel [68]. In practice, panels are often adapted, depending on the specific research question. A cautious approach would be to train ImmuNet from scratch for each panel. This would be laborious but may be feasible given the relative ease with which large numbers of annotations can be collected—using our internal tooling, a trained operator can typically annotate a few hundred cells per hour. However, when only one or two markers change, it may be effective to pool the data, especially if the alternative surface markers are of the same type (e.g. if one membrane marker is replaced by another). Similarly, if some markers turn out to be unreliable in certain samples because of unspecific staining, one can still pool the data with other samples where the same marker is used but zero out the unreliable channel. In future research, we would like to investigate to what extent transfer learning strategies [69] could be employed to mix different channels in a more flexible manner. However, this may not be straightforward for mIHC data given the spillover effects between adjacent channels frequently seen in such data and might be a more fruitful avenue for other multiplex imaging techniques that are less affected by spillover such as CyTOF. Architectural improvements may also be of interest; for instance, based on the results of a recent multimodality cell segmentation challenge, a transformer architecture seems worth exploring [14].

Our T cell panel is designed to identify known cell types, and our data contained annotations for every cell type. However, ImmuNet is not inherently limited to this scenario. Since the network generates a pseudomarker output for every channel, it is, in principle, capable of detecting cell types that did not occur in the training data—such as rare cells expressing previously unobserved combinations of markers. In this paper, we did not evaluate to what extent this kind of generalization to new cell types works in practice, but this would become essential for imaging methods that can combine a large number of cellular markers, such as CODEX with a capacity of up to 60 markers [4, 70]. With such high-dimensional data, annotating every channel for every cell and including explicit examples for every possible cell type would no longer be feasible. It would therefore be interesting to determine whether ImmuNet can be effective in such a scenario, which would probably require modifying the training procedure to predict the expression of single markers separately. If pre-trained on a large and diverse enough dataset, such an approach could be capable of generalizing to unseen combinations of markers during inference. On the other hand, we anticipate that robustness to dataset- or technique-specific staining artifacts might be difficult to achieve. Alternatively, problems with rare cell types could be mitigated by gathering more annotations with a human-in-the-loop approach—we already implemented this when building our model for a dendritic cell panel [68]—or by importance sampling.

Generating synthetic data for pre-training a model is another promising way to improve the generalizability, robustness, and scalability of ImmuNet. Synthetic datasets could either contain a very diverse set of cell shapes, staining artifacts, and phenotypes, or they could be made as similar as possible to the real images generated by a given pipeline, possibly with an increased occurrence of rare phenotypes. Methods to generate plausible histopathology images have recently been proposed [71, 72], but the similarity to real tissue may be harder to achieve for more complex multiplex data. Another promising direction for research that utilizes synthetic data is to further investigate the performance limits of machine learning models depending on artifacts present in images. This would be an extension of the analysis of simulated data we presented in this article. The Cellular Potts framework can be modified to simulate the different degrees of artifacts, such as channel crosstalk and steric hindrance, and then its output can be used to check the robustness of different models in cell detection, phenotyping, or segmentation.

In summary, ImmuNet is a simple but effective and fast ML pipeline for cell detection and phenotyping in multiplex imaging; for example, we have already used ImmuNet to analyze melanoma [73], lung cancer [31], prostate cancer [32], bladder cancer [33], rectal cancer [74] and other cohorts. We have also successfully optimized ImmuNet for a panel designed to detect dendritic cells [68]. Although we developed and tested ImmuNet for FFPE mIHC data, it should also be applicable to other multiplex imaging systems such as CyTOF, CODEX, and NanoString. We hope that the ImmuNet pipeline will help researchers generate more reliable phenotype maps of immune cells in tissue samples as a robust basis for exploratory, diagnostic, and prognostic applications of multiplex imaging technologies.

## Supplementary Material

bpae094_Supplementary_Data

## Data Availability

The immunohistochemistry images used to train the model and the corresponding annotations are publicly available for download on Zenodo [75]. These data can be used to test the provided model training and inference scripts (see below).

## Code availability

The source code of the ImmuNet model, including scripts to train the model and to perform inference, is available at our GitHub repository at https://github.com/jtextor/immunet under the MIT license. The model is implemented in Python 3.6.9 and Tensorflow 1.14.0. The final trained model is available on Zenodo [75]. The code used to perform the analyses reported in this paper is available on Zenodo [76].
