# Supplementary figures and images for: ImmuNet: a segmentation-free machine learning pipeline for immune landscape phenotyping in tumors by multiplex imaging
Source: Biol Methods Protoc. 2024 Dec 20;10(1):bpae094. doi: 10.1093/biomethods/bpae094 (PMC11769680; doi:10.1093/biomethods/bpae094)

**A**

Annotations

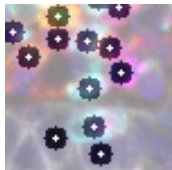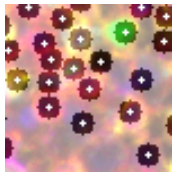**B**

Proximity

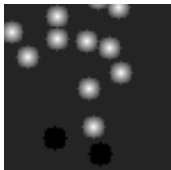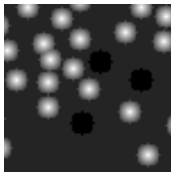 $\psi$ CD3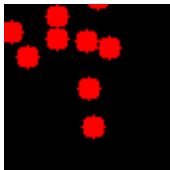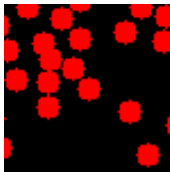 $\psi$ CD20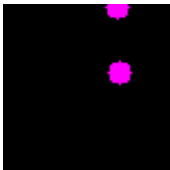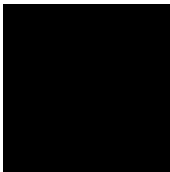 $\psi$ FOXP3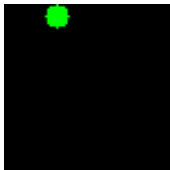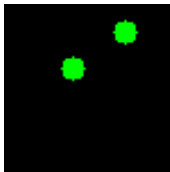 $\psi$ CD8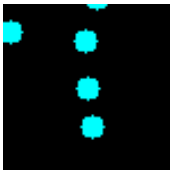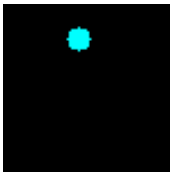 $\psi$ CD45RO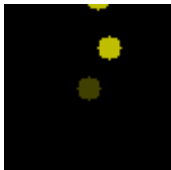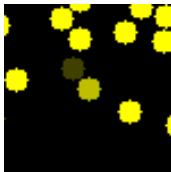

Supplement: bpae094_Supplementary_Data [file bpae094_supplementary_data.zip › supp-figure-5.pdf]

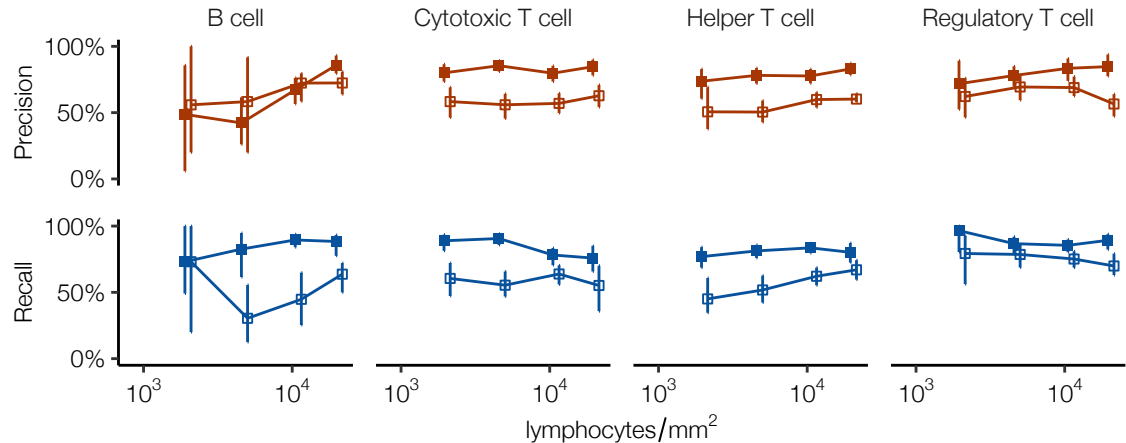

Supplement: bpae094_Supplementary_Data [file bpae094_supplementary_data.zip › supp-figure-2.pdf]

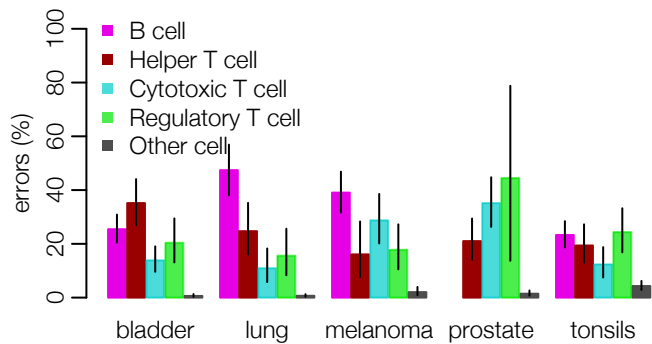

Supplement: bpae094_Supplementary_Data [file bpae094_supplementary_data.zip › supp-figure-1.pdf]

**A**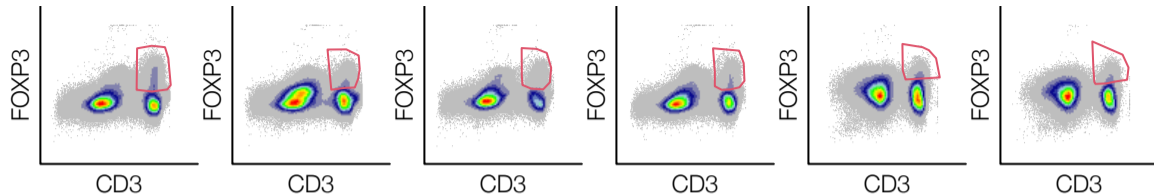**B**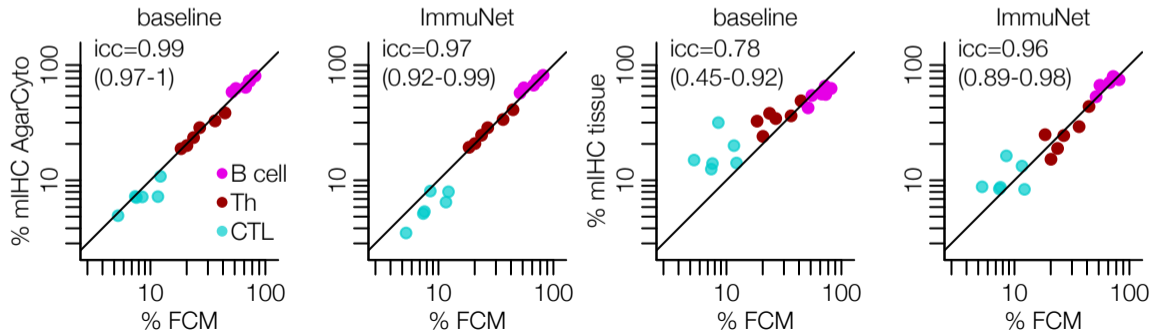

Supplement: bpae094_Supplementary_Data [file bpae094_supplementary_data.zip › supp-figure-3.pdf]

**A**

■ tumor ■ stroma

● lymphocytes

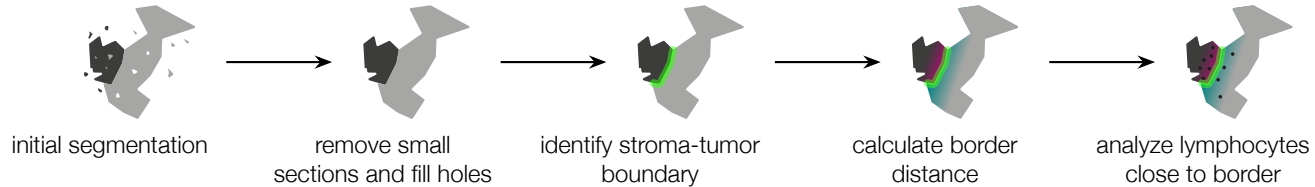**B**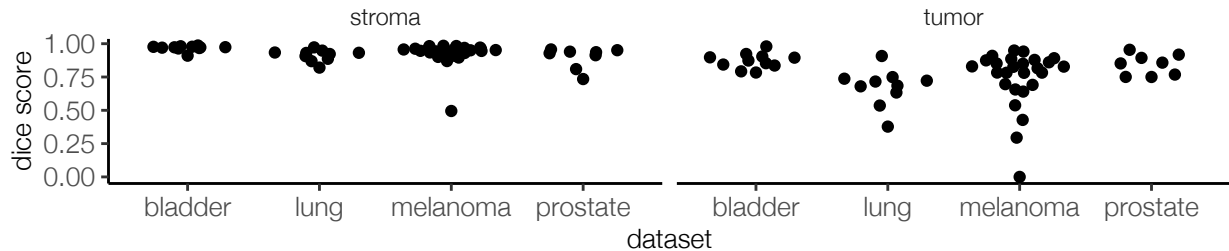

Supplement: bpae094_Supplementary_Data [file bpae094_supplementary_data.zip › supp-figure-4.pdf]
